# Identifying Conservation Successes, Failures and Future Opportunities; Assessing Recovery Potential of Wild Ungulates and Tigers in Eastern Cambodia

**DOI:** 10.1371/journal.pone.0040482

**Published:** 2012-10-15

**Authors:** Hannah J. O'Kelly, Tom D. Evans, Emma J. Stokes, Tom J. Clements, An Dara, Mark Gately, Nut Menghor, Edward H. B. Pollard, Men Soriyun, Joe Walston

**Affiliations:** 1 Wildlife Conservation Society Cambodia Program, Phnom Penh, Cambodia; 2 Forestry Administration, Royal Government of Cambodia, Phnom Penh, Cambodia; 3 Wildlife Conservation Society Global Conservation Program, Bronx, New York, United States of America; 4 Imperial College London, Division of Biology, Silwood Park Campus, Ascot, Berks, United Kingdom; 5 Institute of Zoology, Zoological Society of London, Regent's Park, London, United Kingdom; 6 Department of Zoology, University of Cambridge, Cambridge, United Kingdom; Texas A&M University, United States of America

## Abstract

Conservation investment, particularly for charismatic and wide-ranging large mammal species, needs to be evidence-based. Despite the prevalence of this theme within the literature, examples of robust data being generated to guide conservation policy and funding decisions are rare. We present the first published case-study of tiger conservation in Indochina, from a site where an evidence-based approach has been implemented for this iconic predator and its prey. Despite the persistence of extensive areas of habitat, Indochina's tiger and ungulate prey populations are widely supposed to have precipitously declined in recent decades. The Seima Protection Forest (SPF), and broader Eastern Plains Landscape, was identified in 2000 as representing Cambodia's best hope for tiger recovery; reflected in its designation as a Global Priority Tiger Conservation Landscape. Since 2005 distance sampling, camera-trapping and detection-dog surveys have been employed to assess the recovery potential of ungulate and tiger populations in SPF. Our results show that while conservation efforts have ensured that small but regionally significant populations of larger ungulates persist, and density trends in smaller ungulates are stable, overall ungulate populations remain well below theoretical carrying capacity. Extensive field surveys failed to yield any evidence of tiger, and we contend that there is no longer a resident population within the SPF. This local extirpation is believed to be primarily attributable to two decades of intensive hunting; but importantly, prey densities are also currently below the level necessary to support a viable tiger population. Based on these results and similar findings from neighbouring sites, Eastern Cambodia does not currently constitute a Tiger Source Site nor meet the criteria of a Global Priority Tiger Landscape. However, SPF retains *global* importance for many other elements of biodiversity. It retains high *regional* importance for ungulate populations and potentially in the future for Indochinese tigers, given adequate prey and protection.

## Introduction

Within living memory the dry forests of Indochina (Cambodia, Viet Nam and Lao PDR) were among the “great gamelands of the world” as they supported aggregations of ungulates, including Asian elephant (*Elephas maximus*), wild cattle (gaur *Bos gaurus*, banteng *Bos javanucis*, kouprey *Bos sauveli*, wild water buffalo *Bubalus arnee*) and deer (e.g. sambar *Rusa unicolor* and Eld's deer *Rucervus eldii*) impressive enough to rival those found on African savannas [1]. These forests also purportedly supported high densities of large carnivores, including tiger (*Panthera tigris*), leopard (*Panthera pardus*) and dhole (*Cuon alpinus*) [2], [3].

Conflict and economic development have wrought profound changes in recent decades and, although extensive areas of intact habitat remain (especially in Cambodia), ungulates and carnivore densities are typically perceived as being severely depressed, with many local extinctions apparently occurring even within designated protected areas [4]–[6]. All Indochinese large ungulates other than wild pig (*Sus scrofa*) and red muntjac (*Munticaus muntjak*) are now globally threatened [7] and the kouprey (*Bos sauveli*) is considered most likely extinct [8]. Of the large carnivores, tigers are thought to have now disappeared from most of their former range across Asia [9], [10] and the Indochinese tiger (*P. tigris corbetti*) is predicted to be the next sub-species to be extirpated [11]. The “empty forest syndrome” [12] is becoming an increasingly pervasive reality for the region [13], [14].

Wild cattle, wild pig and deer comprise the primary prey base for top predators such as tiger, leopard and dhole [15], [16] and high ungulate densities have been found to be a critical determinant of viable tiger populations [16], [17]. Retaining ungulate and carnivore communities also has important ecological implications beyond the intrinsic value of each species. For example, ungulates are instrumental in processes such as seed dispersal, nutrient cycling and succession, and they fulfill a key role in the maintenance of habitat structure, composition and dynamics [18].

The impoverished status of Indochina's forests today is generally attributed principally to high levels of illegal hunting, predominantly to supply local, regional and global markets with meat, trophies and other body parts [4], [13], [19]. Large-bodied mammals such as carnivores and ungulates are known to be especially vulnerable to extinction due to their intrinsically lower rates of population increase and the fact that they are disproportionately targeted by humans [14], [20].

As part of its post-war reconstruction since 1992 Cambodia has demonstrated considerable commitment to biodiversity conservation with approximately 24% of the country now designated as protected areas [21]. With suitable investment the opportunity exists to recover large, diverse and robust mammal populations across extensive conservation landscapes. There is growing recognition that conservation efforts should be guided by wildlife monitoring programs that yield rigorous information on population abundance, distribution and responses to specific conservation interventions [22]–[24]. Such information is a prerequisite for determining whether conservation initiatives are achieving their stated objectives and prioritizing investment accordingly [24]–[26]. Across most of Indochina such programs are lacking. This is largely a consequence of the inherent financial, logistical and practical challenges associated with the estimation and monitoring of mammal populations in tropical forests [27], further compounded by the apparent low population densities now prevalent for most Indochinese species of conservation significance.

In this paper we present the results of the first six years of a pioneering program to monitor ungulates and tigers in the Seima Protection Forest (SPF) in eastern Cambodia. SPF forms part of a Global Priority Tiger Conservation Landscape [28] and one of the largest remaining tracts of tiger habitat in Indochina [11]. The paper has four principal components. Firstly, we present the current status and recent population trends of wild ungulates in SPF, obtained using distance-based sampling methods. To our knowledge these represent the most rigorous peer-reviewed estimates for these species in Indochina to date. Secondly, we assess the status of tigers through the application of a suite of intensive field survey methods. Thirdly, we assess the potential of the ungulate population to support recovery of wild tigers. Finally, we consider the implications of our results in the wider context of both ungulate and tiger conservation in Indochina

## Methods

### 2.1. Study Site

**Figure 1:**
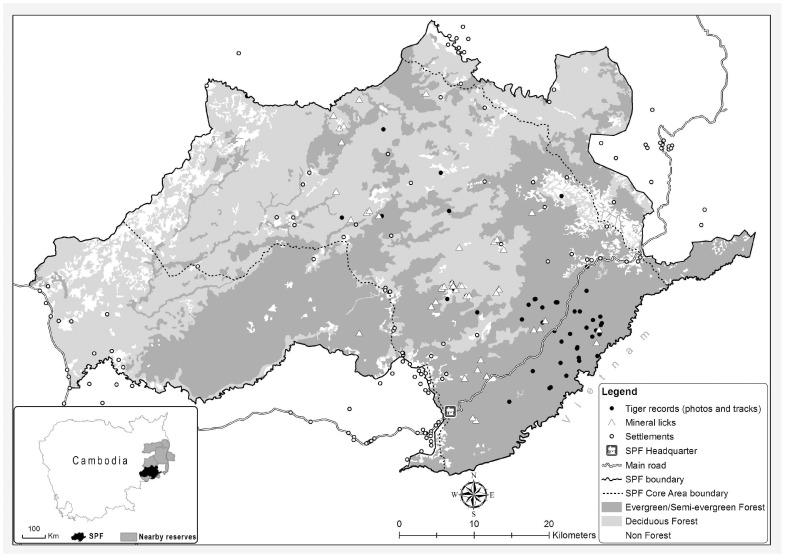
Seima Protection Forest: Main features and all tiger records (2000–2007).

SPF (2927 km^2^ 70–750 m asl) has a tropical monsoonal climate with 2200–2800 mm/year of rainfall and up to 5 dry months per year from December-April [29]. It represents a convergence of the Eastern Plains of Cambodia and the Southern Annamite mountain range and is characterized by a complex mosaic of forest types varying from fully deciduous to almost fully evergreen. The additional presence of areas of open grassland, numerous permanent water sources and mineral licks has resulted in a highly productive landscape (Fig. 1). In 2000, surveys in SPF identified it as a site of high regional conservation priority for biodiversity in general, and for carnivore and ungulate species in particular [29], [30]. These surveys yielded the first ever photographs of wild tigers in Cambodia, and evidence of a largely intact assemblage of tropical forest ungulates [30]. Qualitative assessments strongly suggested tiger and prey populations had recently undergone sharp declines and that densities were depressed compared to natural levels [4], [5], [31]. However, although no empirical data were available, it was believed that these populations had been less severely affected than those at most other sites in Indochina, and populations were deemed to have high recovery potential [4], [5], [31].

Since 2001, the site has been managed by the Forestry Administration (FA) supported by the Wildlife Conservation Society (WCS). The principal threat to large mammals in SPF is hypothesized to be direct hunting, and, in the case of large carnivores, the hunting of prey species [29], [32]. Habitat loss, degradation and disturbance are also likely to be increasingly significant [29]. The primary strategy to address these threats is through direct law enforcement [29], [32]. Thus, management interventions in SPF have included a strong direct protection component aimed at relieving illegal hunting pressure on targeted species by means of anti-poaching patrols. Additional interventions implemented include policy support, community natural resource management and the development of alternative livelihoods [29].

From the earliest stages of this work a monitoring program was developed to quantify the response of wildlife to management interventions and to measure progress towards conservation objectives [24], [33]. It covers tiger, seven ungulate species, six primates and one bird (green peafowl *Pavo muticus*). Here we report the results for tiger and for those ungulate species that form part of their regular prey base (i.e. all except elephant).

### 2.2. Ungulate surveys

#### 2.2.1. Survey design

Line transect-based distance sampling methods were used to estimate ungulate density in SPF [34]. Distance sampling addresses two of the most problematic aspects of animal abundance estimation; spatial sampling and variation in detection probability. This allows for the generation of unbiased density estimates which can be compared across time and space. Survey design in SPF proceeded in two phases: Phase 1 (2005–2007) when designs were tested with low survey effort and Phase 2 (2008–2010) with an improved design, employing higher effort by more skilled field teams.

**Figure 2:**
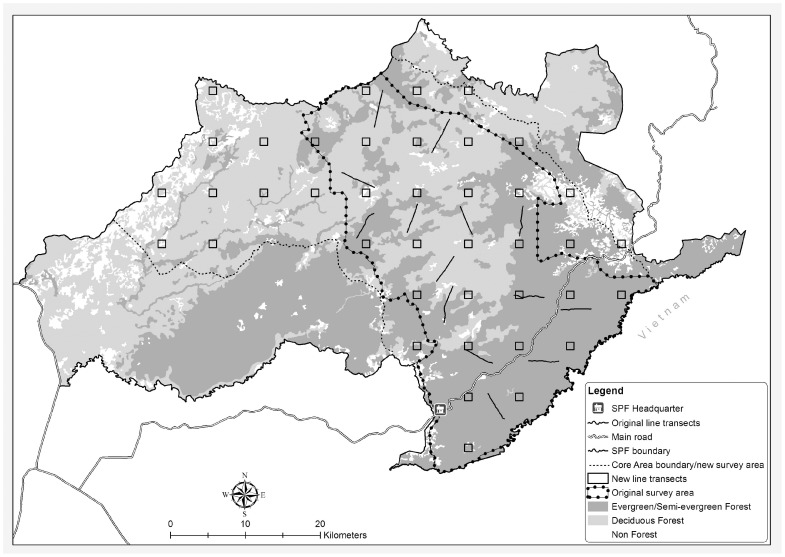
Original line transect survey design (2005–2008) and new design (2010).

During 2005–2007 14 transects, each 3–5 km in length, were monitored within a 1086 km^2^ survey area encompassing the most important habitat for large-bodied mammals within the site [33]. Transects were placed randomly, with stratification by broad forest type (Fig. 2). In 2005 and 2006 each of the 14 transects were surveyed twice per season (133 km total) and in 2007 they were surveyed three times (170 km total). As a consequence of the low survey effort during this period encounter rates for ungulate species were extremely low and variable

In 2008 sampling effort was increased eight-fold as each of the 14 transects was surveyed between 32 and 34 times, twice daily over a three-four day period (1359 km total). In 2010 this level of effort was maintained while the number of spatial replicates was increased and the survey area was expanded. The new design consisted of 40×4 km closed circuit transects, which were established across an enlarged 1807 km^2^ survey area corresponding to the SPF core zone (Fig. 2). Transect placement was systematic, with a random starting point, which ensured representative spatial sampling of the entire SPF core zone. With the revised sampling design each of the 40 new transects was walked a total of ten times, twice daily over five consecutive days (1600 km total). No surveys were conducted in 2009.

#### 2.2.2. Data collection

Field protocols were consistent across all years and based on standard line transect methodology for large herbivores [34], [35]. Transects were walked in the hours just after sunrise and those just preceding sunset by survey teams consisting of two trained observers only. Walking speed was between 1 and 2 km/hr. For each group of target species encountered the following information was recorded: location (UTM co-ordinates), species, size of cluster (i.e. group number), observer to cluster sighting distance, compass bearing to cluster centre, and compass bearing of the transect line. The latter three pieces of information were used to calculate the perpendicular distance of the centre of the observed cluster from the line. Garmin GPS units were used to record UTM coordinates, laser rangefinders to measure distances and sighting compasses to take bearings.

#### 2.2.3. Data Analysis

Distance software version 6.0 [36] was used to estimate encounter rates, detection probability, cluster density and abundance, and animal density and abundance of all target species. Prior to analysis, field data were checked for evidence of evasive movement before detection, and potential “rounding” and “heaping” errors [34]. Data were truncated to remove outliers and improve model-fitting. The model which best described the detection process was selected on the basis of Akaike's Information Criterion (AIC), although the goodness-of-fit tests were also considered, and the fit of proposed models to the observed data was examined visually. The methods used to estimate model parameters and to calculate the standard error, coefficient of variation and 95% confidence intervals for each parameter are described in detail in [34]. Analyses were carried out separately for each species, with the exception of wild cattle, where both species were combined due to small sample sizes. As all of the target ungulate species occur in groups, cluster density was estimated first and subsequently multiplied by estimated cluster size to provide an estimate of animal density. In cases where there was evidence of size bias in the detection process (at specified α of 0.15) cluster size was corrected by regression against probability of detection. Density estimates were multiplied by the surface area of the study site to obtain corresponding abundance estimates.

During Phase 1 (2005–2007), low sample sizes (less than the 60–80 observations recommended by [34]) prevented the estimation of annual detection probability and necessitated data pooling across all years (2005–2010). Global detection functions were derived separately for sambar, wild pig, red muntjac and a category combining both wild cattle species. These were then used retrospectively to generate annual population estimates for each species. Such an approach is imperfect in that it assumes a constant detection probability over time but with such low encounter rates this was considered the optimal approach. In Phase 2 (2008–2010), use of the global detection function to estimate annual densities was still required for sambar and wild cattle. For the more abundant species such as red muntjac and wild pig it was possible to estimate both species- and year-specific detection functions in 2008 and 2010. These estimates showed detection probability to be reasonably consistent across time for both species, partially validating the pooling approach for rarer species.

Two separate analyses were conducted for the 2010 data. Firstly, 2010 data were truncated to include only the area sampled between 2005–2008, enabling meaningful comparisons over time. Analysis of the full 2010 dataset was also carried out, to obtain an estimate for the entire core zone where surveys will be replicated in future years.

### 2.3. Tiger Surveys

#### 2.3.1. Survey design

During 2005–2010 several field methods for surveying tigers were applied in sequence. As more information became available the objective changed from determining distribution to estimating population densities to reliably establishing presence of any remaining individuals.

#### 2.3.2. Camera Trapping

Ad-hoc camera-trapping in SPF has been conducted on an on-going basis since 2000 and effort has been highly variable in terms of intensity of effort and camera-placement. Camera-trapping was initially carried out to investigate the presence or possible absence of various cryptic species during the first surveys of wildlife in the area. All camera-trapping to date has focused on the southern and central sections of the site, which is where earlier tiger records were concentrated.

During 2005–2007 opportunistic camera trapping was concentrated on focal mineral licks and water sources, which are important sites for key tiger prey species. During 2008–2010, opportunistic camera trapping focused more on trails and dry stream beds, which tiger are known to use preferentially [37], [38].

**Figure 3:**
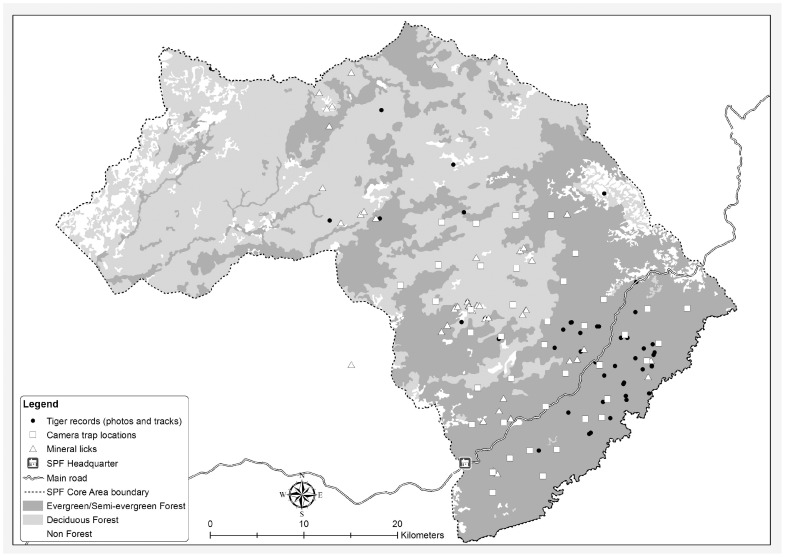
Systematic paired camera-trap survey (2007).

In 2007, a systematic camera-trap survey was conducted based on the capture-recapture sampling approach developed by [37]. The survey area encompassed 750 km^2^ in the southern part of the site and was sampled in three consecutive blocks. Paired *DeerCam* units were placed in a total of 40 locations, for a period of 20 days per location (Fig. 3). Prior to the installation, topographic maps were consulted and suitable trap locations were selected based on the presence of roads, trails, dry river-beds and other natural funnels in the topography, as well as on the existence of prior tiger records. It was ensured that each camera was a maximum of 5 km from another so that there were no “holes” in trapping effort (*sensu* [37]). Camera-trapping took place over a 72 day period, resulting in a total effort of 820 trap-nights.

#### 2.3.3. Sign Surveys

In 2007, a permanent tiger team was established to identify and search potential tiger “hotspots” for all tiger sign including track, scrapes and scent marks. Members of the tiger team were trained at a site in Thailand where tiger sign could be reliably detected and identified. Tiger hotspots included those areas which are typically used by tigers (mineral licks, dry stream beds and forest trails), and where prey densities were thought to be high and levels of human disturbance low. The sign survey continued throughout the 2007/2008 field season and the team also followed up on any reports of tiger received from community members, law enforcement staff and other sources. The team also enlisted the help of one local former tiger hunter to assist with the survey.

#### 2.3.4. Detection Dog Surveys

**Figure 4:**
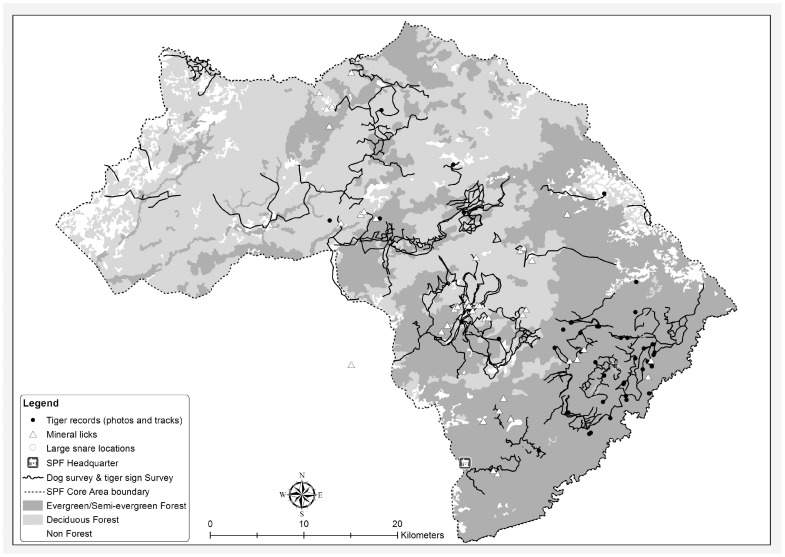
Routes covered by sign survey team and dog and handler team (2008–2010). Previous tiger records and location of large wire snares also shown.

Following the negative results in 2007–2008 surveys there was increasing concern regarding the ability of survey teams to detect animals if they remained only at extremely low densities across a large area, so a specially trained scat detection dog was deployed. Detection dogs search by scent rather than sight which allows them to cover survey areas more efficiently and they can greatly increase the detection rate of survey targets in comparison with human search teams [39], [40]. They are particularly suitable for use in collecting monitoring data on elusive, low density species such as carnivores [39], [40]. In early 2009, a 5 year-old German Wire-haired Pointer arrived in SPF from the Russian Far East where she has been trained and worked as a tiger scat detection dog. From March–June 2009 and January–May 2010, field surveys were conducted by the dog and handler team. The team employed protocols analogous to that used by camera-trap and sign survey teams in that they systematically identified likely tiger hotspots within a pre-defined area and subsequently searched them exhaustively (Fig. 4). The dog and handler team were also on hand to follow up on any local information received on tiger sightings or reports of tiger sign.

## Results

### 3.1. Ungulate Surveys

Estimated densities of ungulates in the SPF core zone in 2010 are shown in Table 1 and density trends during 2005–2010 in the smaller initial survey area are shown in Table 2. No Eld's deer were recorded on the transects.

**Table 1:** Density of ungulates in the expanded survey area 2010^a^.

*Species*	*No. Observations (n)* b	*Density (individs/km2)*	*95% CI lower*	*higher*	*CV%*	*Approximate no. individuals*
**Red Muntjac**	169	1.75	1.22	2.51	18.14	3200 (2200–4500)
**Wild Pig**	52	2.04	1.19	3.49	27.69	3700 (2200–6300)
**Wild Cattle** c ^,^ d	19	0.29	0.11	0.77	50.8	500 (200–1400)
**Sambar** d	6	0.09	0.04	0.23	48.32	200 (100–400)

[CV = % co-efficient of variation; CI = upper and lower 95% confidence intervals].

a These estimates are derived from the full 2010 dataset and are representative of the entire core zone of the SPF where surveys will be replicated in future years.

b Observations are of clusters of animals.

c Data are pooled for the two wild cattle species; comparison of raw encounter suggests approximately equal densities of gaur and banteng but sample sizes are too low to estimate detection probability and density of each species.

d Estimates for wild cattle and sambar are calculated using a detection function derived from data pooled across years 2010 & 2008.

**Table 2:** Density of ungulates in the original survey area, 2005–2010^a^.

*Species*	*Year*	*L (km)*	*n*	*Encounter rate (n/L)*	*Cluster size*	*Density (individs/km2)*	*CV%*	*95% CI lower*	*upper*
**Red Muntjac** b	2005	113	9	0.08	1	1.11	40.5	0.48	2.58
	2006	113	15	0.133	1.1	2.39	25.21	1.4	4.07
	2007	170	25	0.147	1.1	2.55	20.81	1.64	3.95
	2008	1359	134	0.099	1.1	1.75	22.12	1.1	2.79
	2010	920	71	0.077	1.1	1.34	21.45	0.87	2.06
**Wild pig** b	2005	113	3	0.027	2	1.44	54.47	0.48	4.28
	2006	113	5	0.044	1.2	2.4	49.47	0.88	6.51
	2007	170	9	0.053	3.1	2.87	40.55	1.25	6.58
	2008	1359	61	0.045	2.4	1.71	22.91	1.08	2.7
	2010	920	35	0.038	2.8	3.23	33.54	1.68	6.21
**Wild cattle**	2008	1359	28	0.021	3.1	0.61	36.59	0.29	1.27
	2010	960	15	0.016	1.5^c^	0.4	54.82	0.14	1.13
**Sambar**	2008	1359	22	0.016	1.3	0.41	70.1	0.11	1.57
	2010	960	6	0.006	1.2	0.16	46.2	0.06	0.38

[L = total transect length walked; n = number of observations of animal clusters, CV% = percentage co-efficient of variation, 95% CI = upper and lower 95% confidence intervals].

a For this analysis the 2010 data were truncated to include only the area sampled between 2005–2008 (i.e. original survey area) in order to make meaningful comparisons over time.

b Estimates for 2008 and 2010 are based on time-specific detection functions, whereas estimates for 2005–2007 are based on a global detection function derived from pooled data over this period.

c When observations from entire extended survey area in 2010 are included the average cluster sizes is 3.1.

Wild pig and red muntjac densities can be estimated more precisely than other target species. Populations of both appear to have undergone fluctuations over the past five years (Table 2), but the data provide no evidence of sustained declines or increases. Red muntjac in particular appears to have increased and then decreased quite markedly. The difference between the 2010 estimate and that of 2007 is statistically significant (z = −2.008 p<.05) but there are no statistically significant differences between 2010 and any other year (2005 z = 0.416 p>.05, 2006 z = −1.576 p>.05, 2008 z = 0.861 p>.05), suggesting that the 2007 estimate was exceptionally high.

Density estimates for wild cattle exist only for 2008 and 2010 (due to low sample sizes for previous years) and it is not yet possible to examine trends over time. These estimates were obtained using a detection function derived from data pooled across time and species (banteng and gaur) and the precision is low. As data accumulate in future years for both species, the accuracy and precision of the detection function will improve, and can also be applied retrospectively.

The number of sambar observations was low and until sufficient data are available to generate a reliable detection function the figures presented remain provisional for this species. There is also some evidence of evasive movement by sambar before detection, which would violate the assumptions of distance sampling and may result in under-estimates of density and abundance.

Observations of gaur, banteng and sambar were concentrated within the central and southern parts of the site, mainly in areas remote from human influence. Gaur and sambar observations were most frequent in evergreen and semi-evergreen habitat while banteng were typically observed in semi-evergreen and deciduous forest. Wild pig and red muntjac were recorded relatively uniformly on transects across all habitats, and observations were moderately common even in areas subject to high levels of human disturbance. This suggests that these species are more tolerant of anthropogenic pressures than sambar, gaur or banteng.

### 3.2. Tiger Surveys

During 2000–2002 eight camera trap images of at least three individual tigers were captured, with none since. During 2000–2007 over 50 tiger sign records were obtained in SPF, mostly tracks (Fig. 1, Fig. 4). The last confirmed record was a print found in early 2007. Intensive sign surveys during 2007–2008 failed to yield any tiger records. The former tiger hunter who assisted the survey team during 2008 was unable to locate any tiger sign but did lead the team to over a dozen large cable snares, believed to have been targeting tiger (Fig. 4). The detection dog surveys also failed to locate any tiger scat. We conclude that there are currently no resident tigers remaining in SPF, although it remains plausible that transient animals sometimes visit the site.

## Discussion

### 4.1. The status of ungulates in SPF

The core zone of SPF supports approximately 500 wild cattle (gaur and banteng combined), 200 sambar, 3200 wild pig and 3700 red muntjac. Most of these species are also present, albeit in likely smaller populations, outside the core zone. Eld's deer, southern serow and Asian elephant are also present [30], making this one of the most intact assemblages of large ungulates surviving in Indochina. However, kouprey, wild water buffalo and rhinoceros, all presumably once present [30], [41], must have been extirpated before the period of recent surveys.

Despite the fact that estimates for some species are lacking in precision, these data show that the surviving populations of large ungulates at the SPF retain high regional conservation significance. This is particularly true for banteng, currently believed to have a highly fragmented global population of approximately 5000–8000 [42]. In the context of the broader ungulate assemblage it is notable that the SPF elephant population was estimated at 101–139 individuals in 2006 based on dung DNA surveys, and hence is also of at least regional significance [43].

The biodiversity significance of SPF is further enhanced by its position within an unfragmented transboundary conservation landscape of over 15 000 km^2^, encompassing nine reserves (see inset Fig. 1), at least two of which (Phnom Prich Wildlife Sanctuary and Mondulkiri Protected Forest) still also support highly significant populations of large ungulates, including several thousand banteng [44], which is of global significance.

The combined density of large ungulates, excluding elephant, in the SPF core zone is 4.17 km^−2^ (Table 1). Natural densities in Indochinese forests are unknown since we have not traced any published, statistically robust density estimates for these ungulates in Indochina. However, given the historical accounts of ungulate abundance (e.g. [1] and video footage of large herds of wild cattle, no longer seen anywhere in Cambodia) and the apparent suitability of habitat as assessed by experts [4], [30], SPF ungulates densities are likely to be far below the potential carrying capacity of the site. In ecologically similar areas elsewhere in tropical monsoonal Asia, where threats are comparably lower, densities of these species are notably higher than in SPF (Table 3). Although site specific factors may affect the transferability of estimates in the literature, borrowed estimates from other sites can be taken as a broad indicative range of what these habitats could support. The supposition that the current depressed densities in SPF are largely a consequence of past hunting is substantiated by local reports of previously higher densities of ungulates and extremely high levels of hunting during the 1990s [19], [30].

**Table 3:** Published ungulate density estimates (km^2^) for sites ecologically comparable to SPF but with varying levels of protection.

*Species*	*Nagarahole Tiger Reserve, India* a	*Bhadra Tiger Reserve, India* b	*Huai Kha Khaeng Wildlife Sanctuary, Thailand* c	*Taman Negara, Malaysia*	*Seima Protection Forest, Cambodia*	*Source for other sites*
**All ungulates** d	56.1				*4.17*	Karanth et al. 2004
**All ungulates** d		16.8			*4.17*	Karanth et al. 2004
**Red muntjac**	4.2			3.2	*1.75*	Karanth & Sunquist 1992, Kawanishi & Sunquist 2004
**Wild pig**	4.2			4.17	*2.04*	Karanth & Sunquist 1992, Kawanishi & Sunquist 2004
**Wild cattle**			1.8		*0.29*	Srikosamatara 1993
**Sambar**			4.2		*0.09*	Srikosamatara 1993

a Nagarahole has had a long history of effective protection from adverse anthropogenic impacts so these densities can be considered optimal [16].

b Despite being four times higher than those found in SPF ungulate densities in Bhadra are considered to be well below potential capacity due to adverse anthropogenic impacts from several villages located within the reserve [16].

c Although ecologically almost identical, Huai Kha Khaeng has benefited from historically higher levels of sustained protection than SPF, and these ungulate densities are still considered below potential capacity, due to poaching [51].

d Excluding Asian elephant.

Based on the quantitative and qualitative evidence available it seems likely that ungulates underwent steep declines prior to the implementation of conservation activities, but that these declines were halted or slowed during the period under study. Importantly however, the populations of large ungulates in SPF are threatened with a resumption of declines. Law enforcement and wildlife survey teams have recorded high levels of hunting of ungulates in recent years, particularly as access has improved and human migration into the landscape has increased. Guns and snares are the main techniques used and there have been several confirmed incidents of hunting of wild cattle and sambar [29]. While hunting with wire snares persists, the level of gun hunting is undoubtedly lower in the core zone than was observed prior to 2002 (e.g. [19], [30]) when no conservation action was in place and before national gun confiscation campaigns reduced the availability of firearms [29]. However, recent observations suggest that gun hunting is gradually increasing again and the level of hunting pressure is high in relation to the small numbers of large ungulates remaining.

The coarse distribution patterns of gaur, banteng and sambar are broadly consistent with recorded habitat preferences elsewhere in the range of these species [1], [4], [44]. However, not all areas of apparently suitable habitat in SPF are occupied. The evidence suggests that large ungulates have generally persisted in areas characterized by good quality habitat together with some level of protection from hunting, either by virtue of their inaccessibility, or as a result of anti-poaching efforts, or both. This has implications both for the future of these populations within SPF and also for populations in other areas where levels of law enforcement are lower and hunting pressure may be higher.

Estimates for wild pig and red muntjac suggest that populations of both species within SPF are relatively stable and remain moderately abundant despite high hunting pressure. Nevertheless, densities are somewhat lower than would be expected in unhunted sites (Table 3) and while SPF harbours relatively healthy populations of both species, there is potential for further recovery. Further analyses are needed to investigate full the impact of hunting on both large and small ungulate populations, and also to assess the effectiveness of law enforcement at curbing hunting activities.

### 4.2. Implications for tigers

Tigers were reportedly quite common in eastern Cambodia as recently as the 1990s [19], [31] but surveys over the last four years have found no surviving resident population in SPF. This appears to be true for the whole Eastern Plains landscape [11]. Whilst it is conceivable that a few individuals may persist in neighboring protected areas, dedicated surveys, also involving camera-trapping and scat-detection dog surveys, have produced no confirmed evidence of tiger presence since November 2007 (WWF, unpublished data).

Multiple interacting factors are implicated in the rapid loss of the SPF tiger population. Intensive targeted tiger hunting took place in and around SPF during the 1990's [30]. It is also conceivable that the ungulate prey base was depleted to the extent that tiger reproduction and survival rates were lowered [17], thereby further accelerating declines. In the early 1990's it was estimated that 100 to 200 tigers a year were being exported from Cambodia through wildlife markets in Phnom Penh and on the Thai border, with most of the animals reportedly brought in by soldiers posted to the more remote areas of the country [45]. However, the Forestry Administration's Wildlife Protection Office documented a pronounced decrease in poaching records from sites across the country; from 85 poached animals in 1998, to one in 2001, to zero in 2004 [41]. The apparent sharp decline in poaching levels may have been indicative of a final crash in tiger numbers after 15 years of exceptionally high hunting pressure countrywide [11], [41]. By the time conservation interventions were first implemented in SPF in 2002 it was known that the tiger population there was small, but it was believed to hold the greatest potential for recovery of any site in Cambodia. In retrospect, the individuals photographed in SPF may have represented a remnant population with little hope of recovery given the conservation resources available, the escalating threats they were to face over the coming years and the level of investment now known to be required to secure tiger populations [10], [46].

Given the absence of a resident tiger population, we contend this landscape does not constitute a ‘source site’ [10] and no longer meets the criteria for designation as a Global Priority Tiger Landscape [17]. The SPF no longer receives any tiger-specific funding for conservation activities, and tigers are not currently a management priority. Nevertheless, as the only large (>10,000 km^2^) block of dry forest habitat available for tigers anywhere in Southeast Asia, the landscape retains exceptional national and regional importance and remains a potential recovery site for the Indochinese tiger in the future, provided adequate prey and protection for tigers is assured [10]. To restore prey populations, poaching must be eradicated over large areas and other human activities in the vicinity of potential tiger and prey recovery areas must be strictly regulated [11]. Promising initial steps have been taken in SPF but significantly increased long-term investments will be needed to achieve success [29]. An inviolate core area will also be essential if tiger re-introduction is to be considered [46]. We believe that re-introduction is the only feasible option to restore wild tigers to Cambodia.

Our results enable us to estimate the number of tigers that current and potential future ungulate populations in SPF could support. The current prey base is *c.*7500 animals. Using Karanth et al.'s 2004 model [16], which assumes an average kill rate of 50 ungulates/tiger per year requiring a base population of 500 ungulates/tiger, the core area currently harbours sufficient prey to support about 15 tigers (i.e. about 5 breeding females). This is well below the recommended minimum of 75 tigers (*c.* 25 breeding females) in a healthy source site [46], [47], but if prey densities in SPF were recovered to an ecologically feasible carrying capacity of 20 ungulates per km^2^ then the 1800 km^2^ core zone of SPF alone could potentially support over 70 tigers.

If similar prey densities prevail across the core areas of the other two key reserves for large ungulates within the proposed tiger conservation landscape the current prey base would be c.23 500 which is insufficient to sustain the recommended minimum of 50 breeding females (*c.* 150 tigers) required for long-term viability [46]. These calculations show that the recovery of prey populations will be a necessary precondition for successful tiger recovery. Given the regional significance of the ungulate populations themselves, their importance to other predators such as dhole and leopard and the likely benefits to many other, co-occurring threatened species [29], this would be a valuable conservation outcome in its own right.

Continued rigorous monitoring of prey is a crucial part of such an effort, otherwise managers are ‘flying blind’. Repeating surveys on an annual or biennial basis and progressively improving the precision of estimates will identify population trends and allow the testing of hypotheses about the driving factors. The long history of monitoring in SPF also makes this a key regional demonstration and training site, and, along with the recently established program in adjacent sites [44], the only quantitative benchmark that exists regarding the numerical status of ungulate populations anywhere in Indochina.

### 4.3. Broader implications for the conservation of tigers and ungulates in Indochina

SPF is one of the better protected reserves for large ungulates in Indochina, with active law enforcement in place since 2002. Thus, the low densities evident here may imply even lower numbers at many other sites in the region and should heighten concerns regarding the vulnerability of Indochina's remaining large mammal populations. Furthermore, whilst it has long been established that not all suitable forest tracts remaining across Asia are occupied by tigers [10], [17], [48] more rigorous scrutiny of areas where viable tiger populations are currently assumed to persist may reveal extremely low densities and even absences, as is reported in this paper, and was found by [49], [50]. It is often assumed that large forest blocks ‘must’ have a few wily tigers hanging on but defying detection; we suggest that this is true less often than conservationists might wish.

Empirical data must be made available to distinguish conservation successes from failures and prioritize conservation investment accordingly [25], [26]. Without scientifically defensible data on which to base a reliable assessment, the wider status of tiger and prey populations in Indochina remains little more than speculation [48]. Our findings underline the acute need for improved population estimates and trend data for large mammals from key sites in Indochina, and re-emphasize the need for urgent remedial conservation measures in this region. Our results also demonstrate that not only can statistically and biologically robust monitoring methods be applied when challenging conditions prevail, but that they can also provide a solid scientific foundation for pragmatic conservation strategies.
